# Editorial: Human-centered artificial intelligence in interaction processes

**DOI:** 10.3389/frai.2025.1597763

**Published:** 2025-04-15

**Authors:** Maria Chiara Caschera, Patrizia Grifoni, Francesca Cordella

**Affiliations:** ^1^Institute for Research on Population and Social Policies (IRPPS), National Research Council (CNR), Rome, Italy; ^2^Research Unit of Advanced Robotics and Human-Centred Technologies, Campus Bio-Medico University, Rome, Italy

**Keywords:** AI based Human-Robot Interaction, trustworthy and explainable AI, intelligent human-machine collaboration, co-design and AI, collaborative methods and AI, multimodal interaction and AI, human-centered design methodology and AI

Human behavior and communication are inherently complex. This complexity is mirrored in the interactions between humans and machines, which are influenced by the characteristics of communication and human behavior. Humans can convey intricate messages using various interaction modalities, such as voice, gestures, speech, and gaze. These interactions are often driven by emotions that both shape and are shaped by thoughts and actions. Human-centered AI focuses on understanding and addressing the complexities of human-machine interactions. Its goal is to enhance human capabilities by providing intelligent technologies that are informed by human input. Additionally, it learns from human cognitive abilities, aiming to shift some of the cognitive load from humans to technology itself.

This is achieved through methods and algorithms that learn from human input and collaboration, continuously improving by understanding human behavior, emotions, and language. The term Human-Machine Interaction (HMI) encompasses both Human-Computer Interaction (HCI) and Human-Robot Interaction (HRI). The goal is to create effective, efficient, safe, reliable, natural, and intuitive interaction between humans and machines. To achieve a human-centered interaction process, several key aspects must be taken into account. Firstly, the system should be equipped with multimodal interfaces that can be easily adapted to specific contexts, user needs, and preferences, making HMI as natural and intuitive as possible. Secondly, AI should have the capability to operate in dynamic, non-deterministic, and partially unknown environments. Thirdly, it is essential to foster mutual understanding and learning to ensure transparent and explainable interactions. Lastly, systems should be designed to recognize and respond to human gestures, speech, and other non-verbal cues in a way that feels familiar and comfortable to humans.

Having a comprehensive understanding of the design and development processes of current AI solutions is essential for shaping future AI-based systems. The introduction of human-centered AI aims to bridge the gap between machines and humans, making AI genuinely useful in enhancing human capabilities. This approach prioritizes users by considering their needs, contexts, and expectations while adapting to changes in their interaction behaviors. Human-centered AI is complex and encompasses various factors such as social and cultural behaviors, users' abilities, preferences, and limitations. As a result, it requires the involvement of users in the design process, learning from them, and collaborating to create an accessible, effective, and sustainable interaction paradigm.

The goal has been to provide an overview of AI techniques applied to human-machine interaction (HCI and HRI), with a particular focus on a human-centric approach. This Research Topic has explored the latest challenges in AI-driven systems that interact with humans. It aims to identify current advancements in the field to achieve more effective and reliable human-machine interaction.

A total of eight contributions were selected for publication within this Research Topic.

The articles in this Research Topic underline the importance of integrating AI thinking into workflows and adopting a human-centric approach for the future development of corporate work environments by exploring the impact of AI tools on the daily tasks of designers in corporate environments, with a focus on the creation and evaluation processes of design briefs (Zhu et al.). Additionally, the role of user-centered and personalized approaches in artificial intelligence has been examined to objectively and quantitatively measure the effectiveness of explainable AI (XAI) systems, particularly in terms of their “information power” (Matarese et al.).

The AI techniques applied to human-machine interaction have been addressed by investigating research on machine learning applications in scanpath analysis for passive gaze-based interaction (Selim et al.). Furthermore, the potential of ChatGPT 4 in the assessment of personality traits based on written texts has been investigated (Piastra and Catellani). In addition, the impacts of auditory and visual feedback from assistive driving systems on drivers have been assessed to enhance the theoretical foundation in the field of automotive user interface design, particularly concerning the design of auditory functions (Zou et al.).

Within this Research Topic, the impact of AI in various sectors of society has been addressed by understanding the factors influencing AI adoption (Ibrahim et al.). The role of AI has been also addressed in the spread of misinformation on social media by investigating on how AI contributes to the creation of deceptive war imagery (García-Huete et al.). Finally, the impacts of AI dimensions on family communication have been analyzed by investigating the multifaceted effects of AI on family communication (Alfeir).

This Research Topic has collected a cross-disciplinary perspective on human-machine interaction and its influence in several contexts.

